# A Novel T-Cell Engaging Bi-specific Antibody Targeting the Leukemia Antigen PR1/HLA-A2

**DOI:** 10.3389/fimmu.2018.03153

**Published:** 2019-01-18

**Authors:** Amanda C. Herrmann, Jin S. Im, Sumedha Pareek, Wilfredo Ruiz-Vasquez, Sijie Lu, Anna Sergeeva, Jennifer Mehrens, Hong He, Gheath Alatrash, Pariya Sukhumalchandra, Lisa St. John, Karen Clise-Dwyer, Dongxing Zha, Jeffrey J. Molldrem

**Affiliations:** ^1^Department of Stem Cell Transplantation and Cellular Therapy, University of Texas MD Anderson Cancer Center, Houston, TX, United States; ^2^Oncology Research for Biologics and Immunotherapy Translation Platform, University of Texas MD Anderson Cancer Center, Houston, TX, United States

**Keywords:** acute myeloid leukemia, bi-specific antibody, cancer immunotherapy, re-directed cytotoxicity, PR1

## Abstract

Despite substantial advances in the treatment of acute myeloid leukemia (AML), only 30% of patients survive more than 5 years. Therefore, new therapeutics are much needed. Here, we present a novel therapeutic strategy targeting PR1, an HLA-A2 restricted myeloid leukemia antigen. Previously, we have developed and characterized a novel T-cell receptor-like monoclonal antibody (8F4) that targets PR1/HLA-A2 and eliminates AML xenografts by antibody-dependent cellular cytotoxicity (ADCC). To improve the potency of 8F4, we adopted a strategy to link T-cell cytotoxicity with a bi-specific T-cell-engaging antibody that binds PR1/HLA-A2 on leukemia and CD3 on neighboring T-cells. The 8F4 bi-specific antibody maintained high affinity and specific binding to PR1/HLA-A2 comparable to parent 8F4 antibody, shown by flow cytometry and Bio-Layer Interferometry. In addition, 8F4 bi-specific antibody activated donor T-cells in the presence of HLA-A2^+^ primary AML blasts and cell lines in a dose dependent manner. Importantly, activated T-cells lysed HLA-A2^+^ primary AML blasts and cell lines after addition of 8F4 bi-specific antibody. In conclusion, our studies demonstrate the therapeutic potential of a novel bi-specific antibody targeting the PR1/HLA-A2 leukemia-associated antigen, justifying further clinical development of this strategy.

## Introduction

Standard of care therapy for newly diagnosed acute myeloid leukemia consists of highly intensive chemotherapy, followed by allogeneic hematopoietic stem cell transplant in those patients who are at a high risk for relapse. Advances in treatment strategies have been slowly progressing with intensified chemotherapy regimens for younger patients, providing more durable remissions. However, older AML patients have not benefitted from these advances as they often cannot tolerate these highly toxic chemotherapy protocols. New, less toxic treatment strategies for AML patients are desperately needed (1), considering that the median age of AML diagnosis is 68 years of age. Targeted therapy has been revolutionary for the treatment of certain leukemias and have recently made an impact on AML patient treatment (2). Clinical trials evaluating FLT3 inhibitors were recently evaluated in a meta-analysis, and showed in combination with chemotherapy, FLT3 inhibitors increased patient overall survival (3, 4). Recently, the re-introduction and FDA approval of targeted therapies such as gemtuzumab ozogamicin (5), a CD33-targeting antibody conjugate, and other novel immunotherapies have expanded treatment possibilities for patients with AML (6). Adoptive T cell therapy with Chimeric Antigen Receptor (CAR) T cells targeting various leukemia antigens such as CD33 and CD123 are currently under investigation through clinical trials (7).

T-cell engaging antibodies form a novel class of immunotherapy. The most well characterized drug within this class is blinatumomab, a single chain bi-specific antibody simultaneously binding CD3 of T cells and CD19 of B cell leukemia that lead to bring T-cells and CD19^+^ targets into proximity. Subsequently,CD19^+^ target cells are lysed by proximal T-cells. This drug has a 33 month relapse free survival for 61% of patients with B-cell acute lymphoblastic leukemia (B-ALL) (8, 9), and has been approved for relapsed/refractory CD19^+^ B-ALL. This novel strategy is currently being evaluated for redirecting T-cells to target known leukemia antigens, including CD33 (10), and oncoprotein WT1 (11), and solid tumor antigens such as EpCAM and CEA (12).

PR1 is a human leukocyte antigen (HLA)-A2 restricted nonapeptide (VLQELNVTV), which is derived from the serine proteases proteinase-3 (P3) and neutrophil elastase (NE), two myeloid azurophil granule proteases that are highly expressed in myeloid leukemia blasts (13). Studies from our group have established the PR1/HLA-A2 complex as an important antigen in myeloid leukemia as well as some solid tumor malignancies (13–15). We have already demonstrated safety of PR1 peptide vaccination in patients with myeloid leukemia (16).

Additionally, we developed a TCR-like monoclonal antibody (8F4) that targets PR1/HLA-A2 and demonstrated that administration of 8F4 led to complete and durable treatment responses of human primary AML in xenograft mouse models (17, 18). Further, 8F4 based chimeric antigen receptor (CAR) T-cells maintained PR1/HLA-A2 specificity and lysed HLA-A2^+^ primary AML blasts (19). Here, we present the development of a bi-specific T-cell engaging antibody, targeting CD3 on T-cells and the PR1/HLA-A2 complex on AML. We show the 8F4 bi-specific antibody specifically recognizes the PR1/HLA-A2 antigen and CD3 T-cell antigen. Next, we show the antibody facilitates targeted T-cell activation and T-cell mediated cytotoxicity in both AML cell lines and primary AML patient samples. This bi-specific T-cell engaging antibody provides a novel immunotherapy to target PR1-presenting myeloid and solid tumor malignancies.

## Materials and Methods

### Cell Lines, Patient Samples, and Antibodies

Cell lines OKT3, Chinese hamster ovary (CHO), T2 (TAP-deficient), Jurkat, J.RT3-T3.5 (Jurkat cell line lacking functional CD3), THP1, U937, and K562 were obtained from ATCC (Manassas, VA, United States). HLA-A2^*^0201-transduced U937 and K562 cells were a gift from Greg Lizee (20). Cell lines were maintained in complete medium RPMI 1,640 supplemented with 10% FBS, penicillin [100 I.U./mL], and streptomycin [100 ug/mL] 37°C in 5% CO_2_. Human AML leukapheresis samples were collected from patients treated at the University of Texas MD Anderson Cancer Center (MDACC) after obtaining written informed consent under protocols approved by MDACC Institutional Review Board. Monoclonal antibodies against human CD5 (clone UCHT2), CD4 (clone RPA-T4), CD8 (clone RPA-T8), CD69 (clone FN50) conjugated to different fluorophores were purchased from BD Biosciences (San Jose, CA, United States). LIVE/DEAD fixable dead cell stain and CellTracker dye were purchased from Thermo Fisher Scientific (Waltham, MA, United States). Mouse anti-His_6_Tag antibodies were purchased from Miltenyi Biotech (San Diego, CA, United States). Polyclonal goat anti-human IgG HRP was purchased from Sigma Aldrich (St Louis, MO, United States). The 8F4 monoclonal antibody was produced at the MDACC Antibody Core Facility as described previously (17). IL-6, interferon gamma (IFN-γ), and tumor necrosis factor (TNF)-α Quantikine ELISA kits were purchased from R&D Systems (Minneapolis, MN, United States). 3,3′,5,5′-Tetramethylbenzidine (TMB) solution was purchased from Thermo Fisher Scientific (Waltham, MA, United States). The PR1/HLA-A2 or CMV-pp65 biotinylated monomers were produced at the Baylor College of Medicine MHC tetramer core facility in Houston, TX. Peptides for both T2 pulsing and bio-layer interferometry were identical and produced commercially by Bio-Synthesis (Lewisville, TX, United States).

### Construction, Expression, and Purification of 8F4 Bi-specific Antibody

First, single light and heavy chain fragment domains from parent 8F4 and OKT3 were conjugated via (glycine_4_-serine)_2_ or (glycine_4_-serine)_3_ linkers, respectively, and subsequent single chain variable fragments of 8F4 and OKT3 were linked via short peptide, GGRGG. 6xHistidine Tag was added to the C-terminus for metal affinity purification. Single chain fragments were linked using PCR methods described previously (21, 22).

Primers used were as follows: 5′Nhe1_sig_Hu8F4_VL: GCTAGCACCACCATG, 3′rev_Hu8F4_VL_G4S: TGATCCGCCTCCGCCTTTGATTT, 5′G4S_Hu8F4_VH:GGCGGAGGCGGATCAGGAGTGCA, 3′rev_Hu8F4_VH_G4S:CCCGCCTGAACCACCTGAAGAGA, 5′G4S_ mOKT3_ VH:GGTGGTTCSGGCGGGCAGGTCCAGCTGCAG, 3′rev_ mOKT3_VH_ G4S:TGATCCGCCTCCGCCTGAGGAGACTGTGAG, 5′G4S_ mOKT3_VL:GGTGGAGGAGGATCTCAAATTGT, 3′rev_ mOKT3_VL_TH_Xho1:CTCGAGTTAGTGATGGTGATGGTGATGACTACCGCGTGGCACCAGGTTTATTTCCAACTT, Universal Linker_ G4S_3: GGCGGAGGCGGATCAGGAGGTGGAGGATCCGGTGGAGGAGGATCT.

The final construct was cloned into the eukaryotic expression vector pcDNA3.1 (Thermo fisher Scientific, Waltham, MA, United States). CHO cells were transfected with expression vector using lipofectamine (Thermo Fisher Scientific, Waltham, MA, United States) according to manufacturer's instructions, and stable transfectants were generated by G418 drug selection following limiting dilution assays. Stable transfectants were grown in roller bottles, and recombinant proteins were purified from culture supernatant using Nickel affinity chromatography as previously described (22). Subsequently, recombinant proteins were subjected to fast protein liquid chromatography (FPLC) on a GE Superdex 75 10/300 GL column (GE Healthcare Life Sciences, Pittsburgh, PA, United States) to isolate monomeric 8F4 bi-specific antibody. Recombinant proteins were electrophoresed to confirm the molecular weight using 10% SDS-PAGE and stained with GelCode Blue Stain Reagent (Thermo Fisher Scientific, Waltham MA, United States). Western blot was performed to confirm the presence of 6xHistidine Tag using anti-HisTag HRP antibody and Pierce ECL western blotting substrates (Thermo Fisher Scientific, Waltham, MA, United States) as described previously (21).

### Flow Cytometry Analysis of 8F4 Bi-specific Antibody Binding

Peripheral blood lymphocytes (PBL) were prepared from collecting non-adherent lymphocytes after 1–2 h incubation of peripheral blood mononuclear cells (PBMC) which had been isolated using Ficoll density centrifugation of buffy coats (MDACC Blood Bank). For 8F4 bi-specific antibody binding of CD3, Jurkat, J.RT3 and human PBL were incubated with various concentrations of 8F4 bi-specific antibody for 45 min on ice, and stained with a secondary anti-HisTag antibody conjugated with PE fluorophore after washing with PBS. For 8F4 bi-specific antibody binding of PR1/HLA-A2, T2 cells were pulsed overnight with 20 μg/ml of various peptides including PR1 wild type (VLQELNVTV), PR1 with sequential alanine substitutions, WT1 (RMFPNAPYL), MART1 (ELAGIGILTV), and control HLA-A2 restricted CMV-derived peptide pp65 (NLVPMVATV) in complete media. After large volume washes, peptide-pulsed T2 cells were stained with 8F4 bi-specific antibody as described above. All samples were stained with a LIVE/DEAD fixable viability dye. All flow cytometry data were collected using a BD FACSCanto II flow cytometer, and analyzed with Flow Jo Software version 10.1 (Tree Star, Ashland, OR, United States).

### Bio-layer Interferometry

The 8F4 bi-specific antibody binding kinetics were assayed by Bio-Layer interferometry (BLI) with a PALL ForteBio Octet 384 RED system (Menlo Park, CA). For 8F4 bi-specific antibody binding to PR1/HLA-A2, the 8F4 bi-specific antibody, monovalent 8F4 Fab, and bivalent 8F4 parent antibody were chemically coupled to the biosensor, and then dipped into various concentrations of PR1/HLA-A2 as analyte from 300 nM down to 3.7 nM or HLA-A2/pp65 at 900 nM as control. For 8F4 bi-specific antibody binding to CD3, recombinant human CD3εδ fusion proteins (biotinylated, Abcam Catalog #ab205994) was captured on a streptavidin sensor chip at a concentration of 200 nM, and 8F4 bi-specific antibody or parent anti-human CD3 (OKT3) antibody were evaluated for binding to immobilized human CD3εδ fusion proteins at different concentrations. Data analysis was performed using 2:1 global fitting with Octet data analysis software.

### Anti-idiotype ELISA

Sandwich ELISA was used to determine 8F4 bi-specific antibody binding to anti-idiotype antibodies generated against the parent 8F4 antibody. First, anti-idiotype antibodies were coated at 1 μg/mL in PBS overnight at 4°C. After washing unbound anti-idiotype antibodies, 8F4 bi-specific antibody, parent 8F4 monoclonal antibody, control CD1d protein containing a C-terminal His_6_Tag (22), and control Trastuzumab monoclonal antibody were incubated for 1 h at room temperature. After washing, anti-HisTag HRP was used to detect 8F4 bi-specific antibody and CD1d protein bound to immobilized anti-idiotype antibodies, and goat anti-human IgG HRP was used for detection of monoclonal antibodies 8F4 and Trastuzumab. TMB substrate activity was measured at OD450 using a Cytation 3 from BioTek and Fisher Scientific (Pittsburgh, PA, United States).

### T-Cell Activation and Redirected Lysis of AML by 8F4 Bi-specific Antibody

AML cell lines U937 and THP1 were pre-stained with a pacific blue (PB) CellTracker dye and co-cultured with effector PBL at an E:T ratio of 2:1 in complete media with increasing concentrations of 8F4 bi-specific antibody in a 96 well plate for 18 h, 37°C/5% CO_2_. For use as a positive control, AML cells were lysed with 0.1% Triton in PBS. Following incubation, supernatant was harvested for use in ELISA to detect secreted IL-6, IFN-γ, and TNFα and cells were analyzed for viability, CD5, CD4, CD8, and CD69 expression on a BD FACSCanto II flow cytometer. Surface CD69 staining (presented as mean fluorescence intensity) was quantified on live CD4^+^ and CD8^+^ cells for evaluation of T-cell activation. For calculation of 8F4 bi-specific mediated cytotoxicity, total cell counts of live target cells were compared to control groups, and calculated as follows: % cytotoxicity (CTX) = 100 × (Target count at 0 nM 8F4 bi-specific antibody—Target count with 8F4 bi-specific antibody treatment)/(Target count 0 nM 8F4 bi-specific antibody—Target counts with 0.1% triton).

Similar co-culture experiments were performed with healthy donor allogeneic PBL and patient AML leukapheresis samples. The HLA testing of patient leukapheresis was conducted at the MDACC HLA typing laboratory. Prior to use in co-culture experiments, frozen patient samples were thawed and allowed to recover overnight in complete medium. Following co-culture with allogeneic healthy donor PBL at a 2:1 E:T ratio for 18 h, cells were stained for viability, CD5, CD4, CD8, CD69, and CD33/CD34 and flow cytometric data was acquired (control groups were included as described above). To assess 8F4 bi-specific antibody mediated T-cell activation, surface CD69 expression was calculated for live, CD5^+^, CD4^+^ CD5^+^, and CD8^+^ CD5^+^ cells as described above. For calculation of 8F4 bi-specific mediated cytotoxicity, total cell counts of live target cells (CD5^−^, CD33/34^+^) were compared to control groups, and % CTX was calculated.

### Statistical Analysis

All statistical analysis was completed with GraphPad Prism version 7.0c (GraphPad Software Inc., La Jolla, CA, United States). *P* < 0.05 were considered to be significant. Dissociation constants and IC50 concentrations were estimated with 4 parameter non-linear curve fitting. Students *t*-tests (unpaired, two sided) were used to determine significance between groups.

## Results

### Generation of 8F4 Bi-specific Antibody

**Figure 1:**
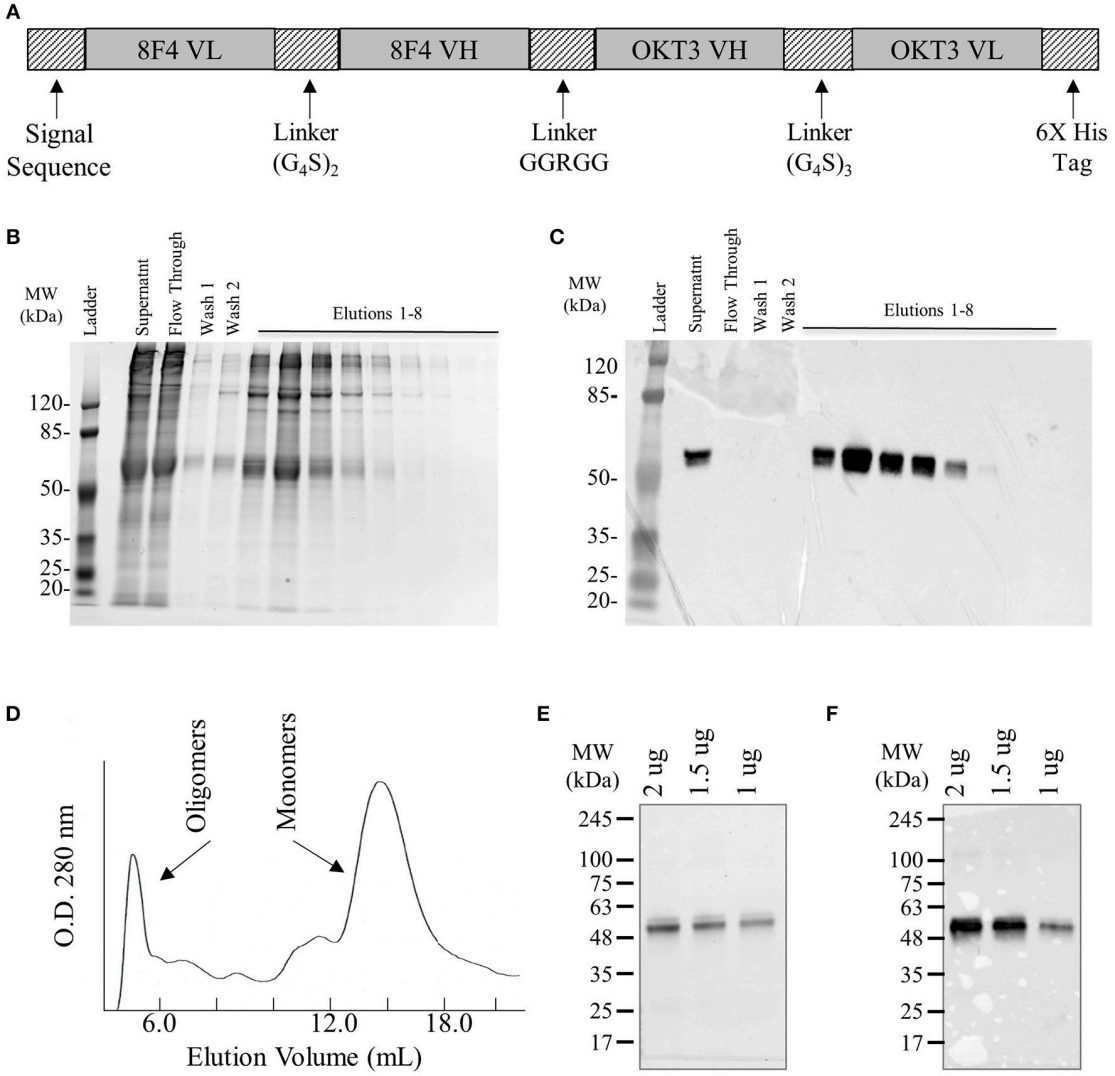
Construction, expression, and purification of 8F4 bi-specific antibody. **(A)** Schematic diagram of the soluble 8F4 bi-specific antibody. Light chain variable fragments (VL) and heavy chain variable fragments (VH) of 8F4 and murine OKT3 are linked in tandem via flexible glycine-serine linkers of either 10 or 15 amino acids. A His_6_Tag was included at the C terminus for purification. **(B)** SDS-PAGE of elution fractions of 8F4 bi-specific antibody from immobilized Nickel affinity chromatography. **(C)** Western blot analysis of 8F4 bi-specific antibody from SDS-PAGE shown in part B, probed with anti-HisTag antibody. **(D)** Fast protein liquid chromatography of soluble 8F4 bi-specific antibody following immobilized Nickel affinity chromatography. **(E)** SDS-PAGE of purified monomeric 8F4 bi-specific antibody. 2, 1.5, and 0.5 μg of 8F4 bi-specific antibody was electrophoresed in a 10% polyacrylamide gel under reducing conditions, and detected using coomassie die. **(F)** Western blot of monomeric 8F4 bi-specific antibody electrophoresed in a 10% polyacrylamide gel under reducing conditions probed with anti-HisTag antibody. Representative images from the multiple independent experiments were shown in **(B–F)**.

The 8F4 bi-specific antibody was constructed by linking single chain variable fragments (scFv) of 8F4 and OKT3 in tandem (Figure 1A). Briefly, heavy and light chain variable fragments from both 8F4 monoclonal antibody and anti-CD3 clone OKT3 were linked with either (glycine_4_-serine)_2_ or (glycine_4_-serine)_3_ linkers. The amino acid link between the 8F4 and OKT3 portions of the bi-specific antibody was kept at 5 amino acids (GGRGG) to encourage flexibility between the 8F4 and OKT3 regions of the construct and allow for optimal simultaneous engagement of target and effector cells. An N-terminal His_6_Tag was included for metal affinity chromatography. First, His_6_Tagged recombinant proteins were enriched using immobilized Nickel affinity chromatography (Figures 1B,C), and subsequently subjected to size exclusion FPLC for purification of monomeric 8F4 bi-specific antibody (Figure 1D). The purity of purified 8F4 bi-specific antibody was confirmed with SDS-PAGE and coomassie staining (Figure 1E) and western blot (Figure 1F). This final product was aliquoted and stored at −80°C. For all experiments presented here, including the SDS-PAGE presented for purity confirmation, were conducted with freshly thawed bi-specific antibody. The final yield of purified 8F4 bi-specific antibody was 400 μg per liter of cell culture supernatants.

### 8F4 Bi-specific Antibody Binding Properties

**Figure 2:**
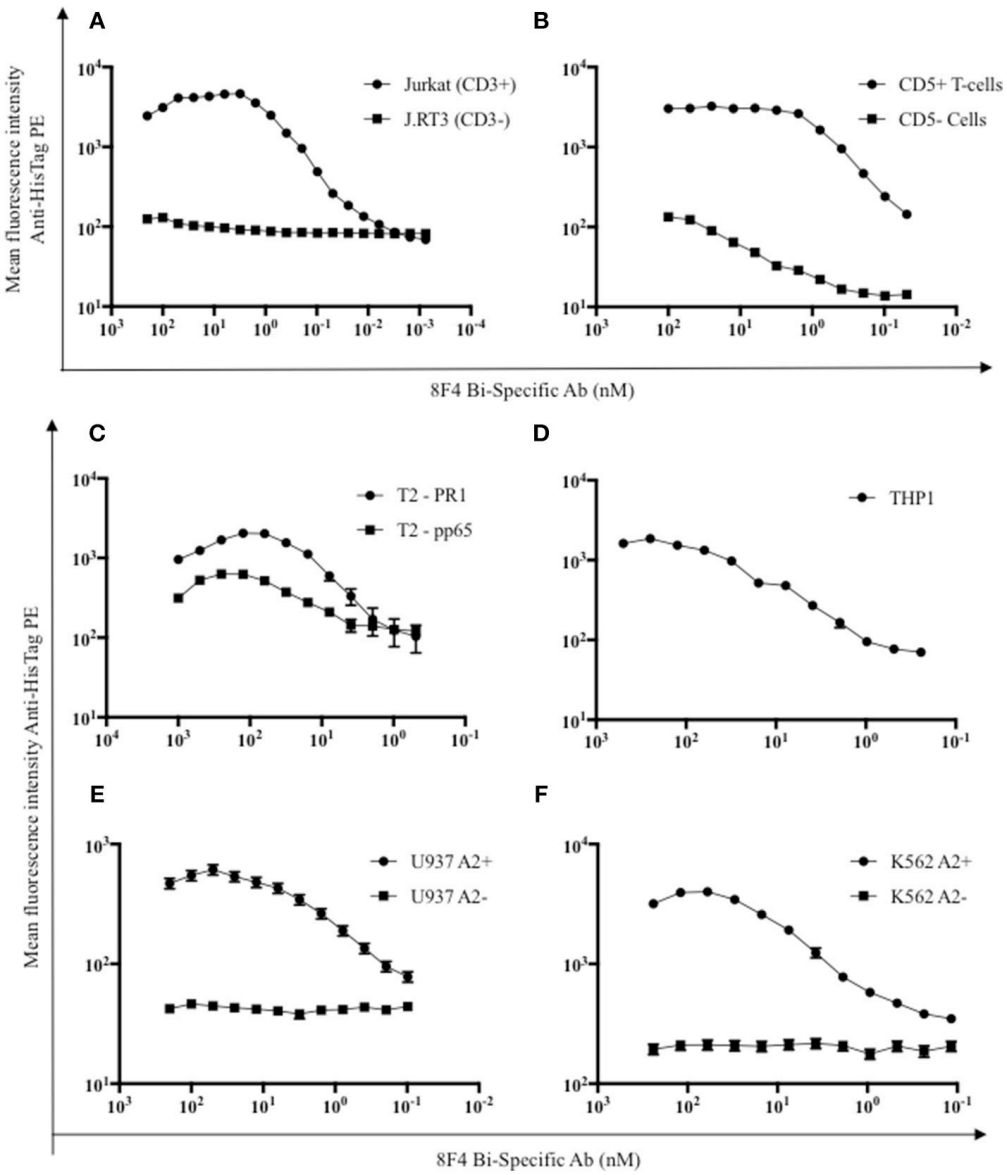
Flow cytometry analysis of target binding specificity by 8F4 bi-specific antibody. Each data point represents the mean of triplicate measures and error bars represent SEM. **(A)** Flow cytometry analysis of 8F4 bi-specific antibody binding of CD3^+^ Jurkat and CD3^−^ (J.RT3) cell lines and **(B)** CD5^+^ and CD5^−^ normal healthy donor peripheral blood lymphocytes in a dose dependent manner. Mean fluorescent intensity is reported. **(C–F)** Flow cytometry analysis of 8F4 bi-specific antibody binding of different PR1/HLA-A2^+^ (T2 PR1, THP1, U937 A2^+^, K562 A2^+^) and control (T2 CMV, U937 WT, K652 WT) cell lines, detected with anti-HisTag PE. Mean fluorescent intensity is reported. These results indicate target-specific PR1/HLA-A2 and human CD3 cell surface binding. Data combined from 2 independent experiments in triplicate or quadruplicate were shown.

First, antigen specific binding of the 8F4 bi-specific antibody was evaluated via flow cytometry. The 8F4 bi-specific antibody showed concentration-dependent binding to Jurkat cells (Figure 2A) and healthy donor CD5^+^ T-cells (Figure 2B) but not to CD3-mutant Jurkat cell line J.RT3 cells. Calculated dissociation constant (K_d_) values were 0.57 and 0.69 nM for Jurkat cells and CD5^+^ T-cells, respectively. This result demonstrates CD3 binding specificity of 8F4 bi-specific antibody.

**Figure 3:**
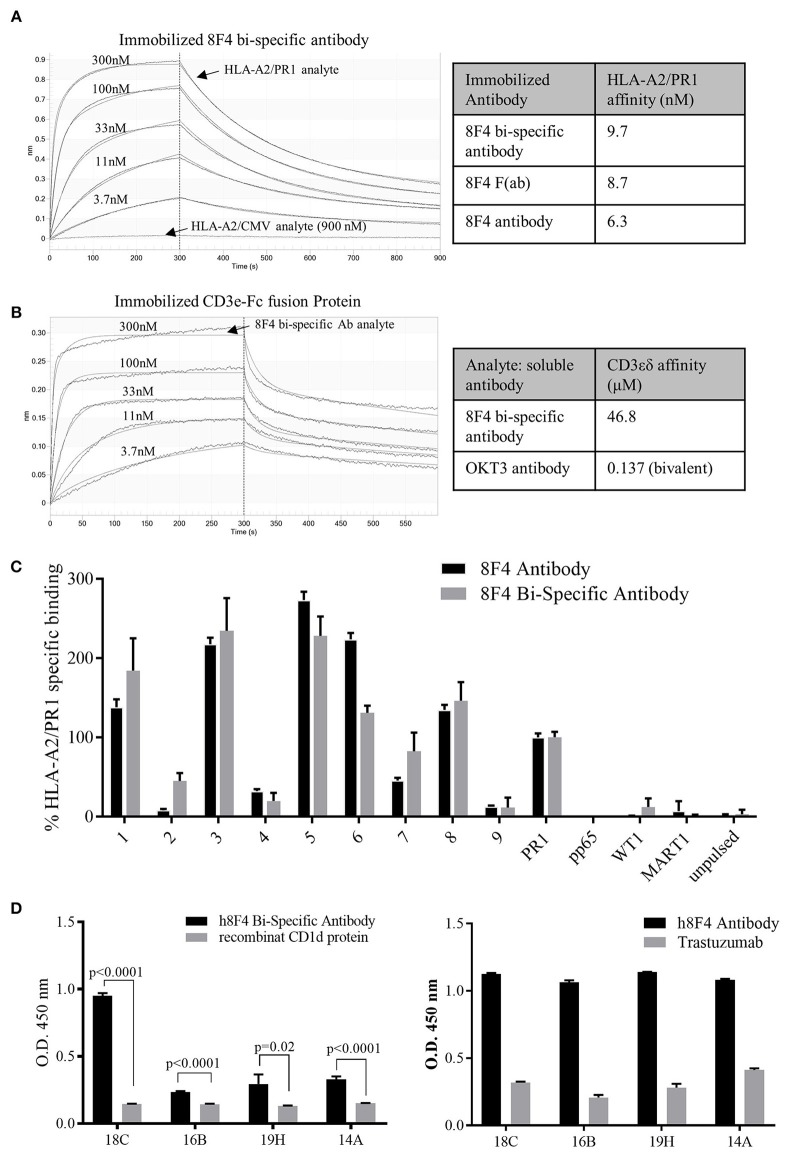
Characterization of 8F4 bi-specific antibody target-specific binding. **(A)** Bio-layer interferometry analysis of PR1/HLA-A2, HLA-A2/CMV monomers binding to immobilized 8F4 bi-specific antibody and **(B)** 8F4 bi-specific antibody binding to immobilized CD3εδ fusion protein. The 8F4 bi-specific antibody showed binding affinity to PR1/HLA-A2 and CD3, with no detectable binding to HLA-A2/CMV. **(C)** Flow cytometry analysis of 8F4 bi-specific antibody and parent 8F4 monoclonal antibody staining of T2 cells pulsed with PR1 peptides containing sequential alanine substitutions, with PR1, CMV-pp65, WT1, MART1 and unpulsed T2 controls. Percent maximum PR1 specific binding is reported. The 8F4 bi-specific antibody showed similar binding pattern to PR1 alanine mutants presented by HLA-A2 compared to parent 8F4 antibody **(D)** Survey of conformational epitopes of antigen binding domain of 8F4 bi-specific antibody. ELISA detection of 8F4 bi-specific antibody and parent 8F4 antibody (with appropriate negative controls) binding to 8F4 anti-idiotype antibody clones. O.D. 450 is reported. Each data point represents the mean of triplicate measures and error bars represent SEM. One representative data of 3 independent experiments were shown.

For evaluation of PR1/HLA-A2 binding specificity, T2 cells, which are HLA-A2^+^ and TAP-deficient, were pulsed with HLA-A2 restricted peptides PR1 and CMV pp65, and 8F4 bi-specific antibody surface binding was quantified (Figure 2C). There was a significant difference (*P* < 0.05) in 8F4 bi-specific antibody binding to T2-PR1 (K_d_ = 4.4 nM) compared to T2-pp65 (K_d_ = 1.2 μM), estimated by 4-parameter non-linear curve fitting setting maximum binding intensity constant for both groups. There was a low intensity biding of 8F4 bi-specific antibody to pp65/HLA-A2 on T2 cells, but not on pp65/HLA-A2 recombinant proteins on solid surface (Figure 3). This can be attributed to the increased 8F4 bi-specific antibody binding avidities to HLA-A2 aggregates expressed on the surface of tumor cells. In addition, 8F4 bi-specific antibody showed strong dose-dependent binding to HLA-A2^+^ AML cell lines THP1 (K_d_ = 30 nM, Figure 2D), U937 A2^+^ (K_d_ = 2.2 nM, Figure 2E), and K562 A2^+^ (K_d_ = 8.4 nM, Figure 2F), but not wild type U937 and K562. THP1 is an AML cell line with endogenous HLA-A2 expression and thus does not have an HLA-A2^−^ control.

Next, Bio-Layer interferometry was used to investigate bio-molecular interactions between the 8F4 bi-specific antibody and specific target molecules to determine affinity. The 8F4 bi-specific antibody had strong interactions with the PR1/HLA-A2 monomer with a calculated K_d_ of 9.7 nM, compared to control HLA-A2/CMV monomer with no reactivity to the 8F4 bi-specific antibody even at high concentrations (Figure 3A). The 8F4 bi-specific antibody interaction with PR1/HLA-A2 was comparable to the parent 8F4 antibody (K_d_ = 6.3 nM) and the 8F4 Fab (K_d_ = 8.7 nM) tested in parallel (sensograms not presented). Further, the 8F4 bi-specific antibody bound the human CD3 epsilon unit (K_d_ of 46.8 μM) as compared the to the bivalent OKT3 antibody (K_d_ = 137nM) (Figure 3B). These results support 8F4 bi-specific antibody PR1/HLA-A2 and CD3 specificity.

To assess similarities in the recognition of target complex PR1/HLA-A2 between the 8F4 bi-specific antibody and the parent 8F4 monoclonal antibody, T2 cells were pulsed with PR1-variant peptides with sequential alanine substitutions and surface staining by 8F4 bi-specific antibody or parent 8F4 antibody was assessed by flow cytometry (Figure 3C). Results showed the 8F4 bi-specific antibody and parent 8F4 antibody have very similar PR1 peptide recognition profiles, with residues at positions 2, 4, and 9 (lysine, glutamine, and valine, respectively) being keys for recognition of the PR1/HLA-A2 complex by both antibodies. Alanine substitutions at these positions reduced 8F4 bi-specific antibody binding by 54, 80, and 97%, respectively. Results also showed minimal surface recognition of other HLA-A2 restricted peptides MART1 and WT1, again supporting PR1/HLA-A2 specificity.

Anti-idiotype antibodies recognize antigen-binding domains of antibodies. Here, we used four anti-idiotype antibody clones specific for 8F4 to assess the antigen-binding domain of 8F4 bi-specific antibody and parent 8F4 antibody (Figure 3D). The 8F4 bi-specific antibody showed significantly higher binding (*P* < 0.02 for all groups) to all four anti-idiotype antibody clones compared to the control CD1d recombinant protein while the parent 8F4 antibody showed strong binding to all 4 anti-idiotype clones as opposed to control Trastuzumab. The 8F4 bi-specific antibody bound the anti-idiotype clones with lower affinity compared to the parent antibody, and this may be due to the monovalent interaction of the bi-specific antibody with its target, as opposed to the bivalent interaction of the parent 8F4. This data again supports the conclusion that the 8F4 bi-specific antibody and parent 8F4 antibody recognize the conformational epitope PR1/HLA-A2.

### The 8F4 Bi-specific Antibody Activates T-Cells and Lyses AML Cells

**Figure 4:**
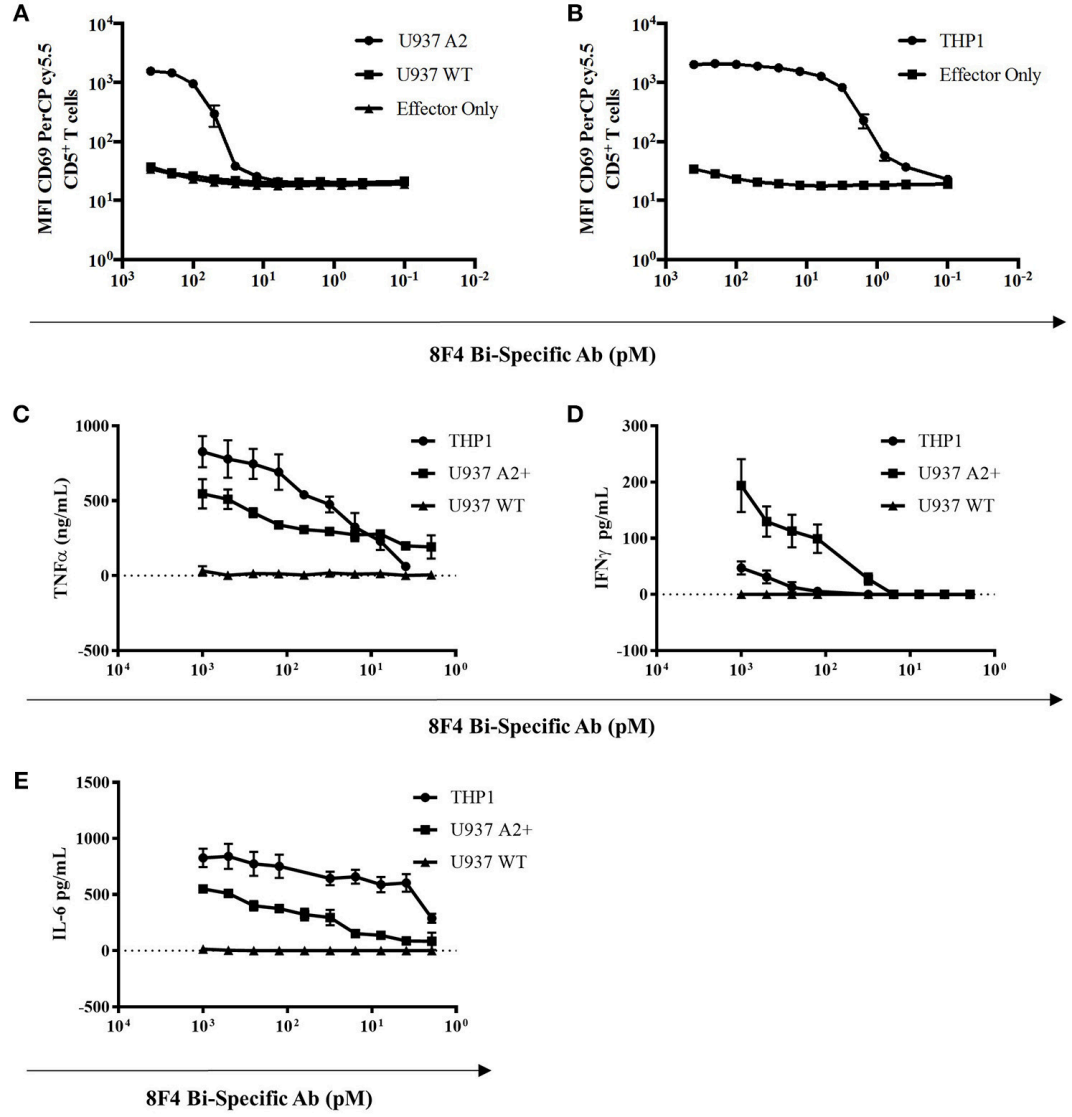
T-cell activation and cytokine production by 8F4 bi-specific antibody. Eighteen hour co-culture experiments combining 8F4 bi-specific antibody with healthy donor lymphocyte effectors and target AML cell lines U937 A2+, U937 WT, and THP1 at an E:T ratio of 2:1 were conducted in triplicate. Bi-specific antibodies led to activation of human PBL in the presence of PR1/HLA-A2 in a dose dependent manner, and activated human PBL produced various inflammatory cytokines only in the presence of 8F4 bi-specific antibody and target AML cells. Flow cytometry assessed surface CD69 on activated T-cells in the presence of target cells as compared to control experiments lacking U937 **(A)** and THP1 **(B)** AML targets. Cytokines from co-culture experiments were then quantified by ELISA for TNFα **(C)**, IFNγ **(D)**, and IL-6 **(E)**. Each data point represents the mean of triplicate measures and error bars represent SEM. One representative data of 2 independent experiments were shown.

Here, we investigated whether the 8F4 bi-specific antibody can activate T-cells in the presence of PR1/HLA-A2^+^ AML cell lines. When co-cultured with AML cell lines U937 and THP1 and 8F4 bi-specific antibody for 18 h, healthy donor CD5^+^ T-cells displayed increased surface expression of the early T-cell activation marker CD69 in a dose dependent manner (Figures 4A,B and Supplementary Figure 2). Of note, low levels of CD69 were also expressed on T-cells incubated in the absence of target cells but only in the presence of high concentrations of 8F4 bi-specific antibody.

In addition, activated T-cells also produced cytokines in response to 8F4 in a dose-dependent manner. Cytokines, including IL-6, IFN-γ, and TNF-α, were all detectable in the co-cultures at the 18 h time point (Figures 4C–E). These results support the ability of 8F4 bi-specific antibody to engage T-cells in the presence of the HLA-A2^+^ AML cell lines U937 and THP1. This engagement leads to activation of the T-cells as evidenced by expression of CD69 and production of cytokines.

**Figure 5:**
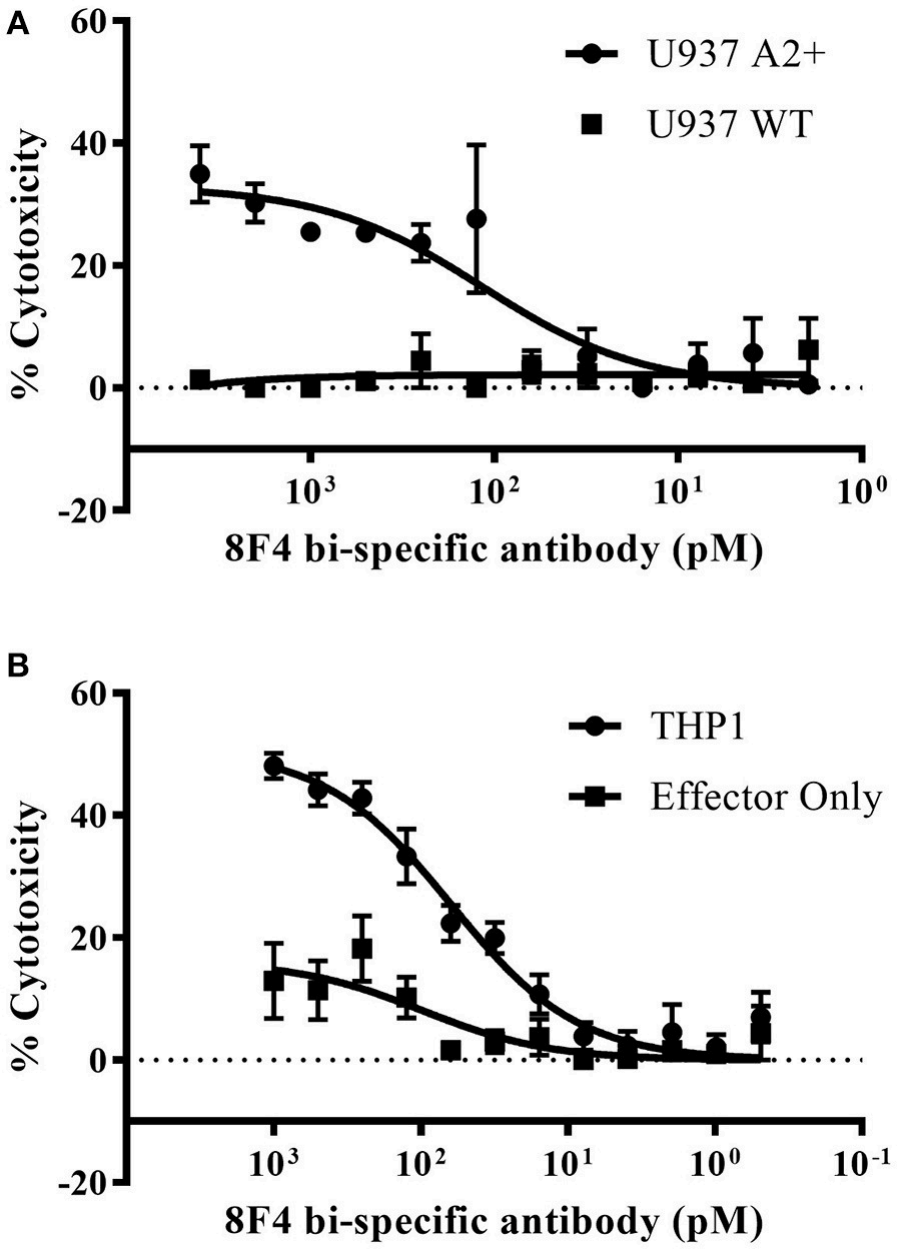
Redirected cytotoxicity of T-cells against PR1/HLA-A2^+^ cell lines. Target AML cells were pre-stained with pacific blue dye and following co-incubation with 8F4 bi-specific antibody and healthy donor PBL at an E:T ratio of 2:1, cells were stained with a fixable live/dead stain. Cytotoxicity calculations were based on total live pacific blue positive cells counts. Calculated % cytotoxicity for co-culture with AML U937 A2^+^ or U937 WT **(A)** and THP1 **(B)** cell lines is presented, based on flow cytometry analysis. These data indicate the 8F4 bi-specific antibody initiated dose-dependent AML-specific cytotoxicity after only 18 h co-culture. Each data point represents the mean of triplicate measures and error bars represent SEM. One representative data of 2 independent experiments were shown.

Next, we determined 8F4 bi-specific antibody mediated cytotoxicity using flow cytometry. Donor PBL were co-cultured with the HLA-A2^+^ AML cell lines U937 and THP1 in the presence of 8F4 bi-specific antibody at various concentrations for 18 h, and viability of AML cells was determined with nuclear uptake of live/dead fixable dye (Figures 5A,B and Supplementary Figure 2). The 8F4 bi-specific antibody resulted in lysis of up to 35% of U937 cells (EC_50_ of 84 pM) and up to 44% of THP1 cells (EC_50_ of 70pM). These results indicate the 8F4 bi-specific antibody can bring about AML cell lysis even after a short incubation time (18 h) and at relatively low 8F4 bi-specific antibody concentrations.

### The 8F4 Bi-specific Antibody Directs T-Cell Cytotoxicity Toward Primary AML Blasts

**Figure 6:**
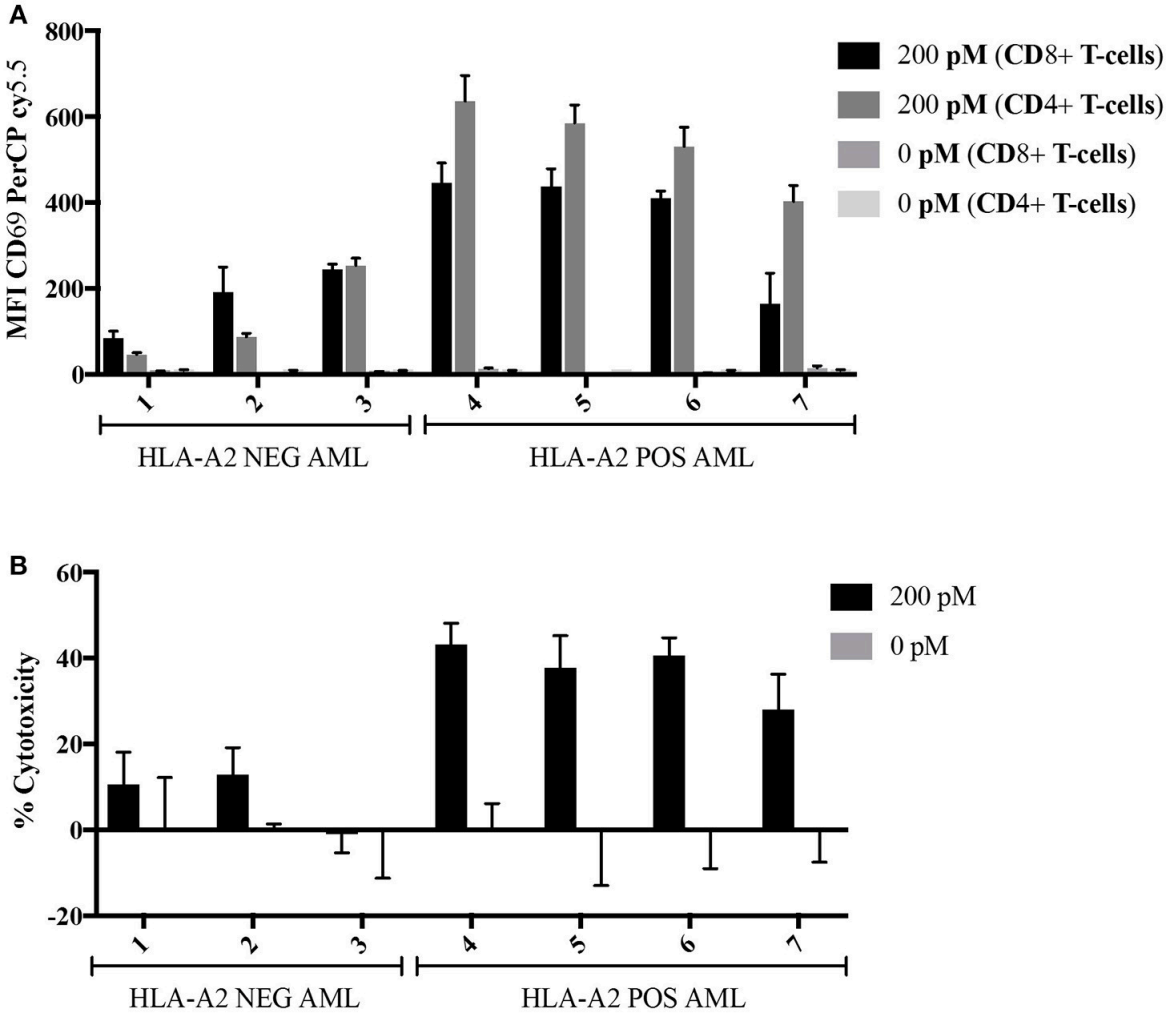
Allogeneic T-cell activation and redirected cell lysis of primary AML patient samples by 8F4 bi-specific antibody. HLA-A2^+^ and HLA-A2^−^ AML patient samples were co-cultured with allogeneic healthy donor PBL at a 2:1 E:T ratio for 18 h in the presence or absence of 8F4 bi-specific antibody. Each bar represents the mean of triplicate measures and error bars represent SEM. **(A)** Summary of T-cell activation for primary patient sample co-culture experiments show increased T-cell activation following co-culture with HLA-A2^+^ patient samples. **(B)** A summary of redirected T-cell cytotoxicity for several HLA-A2^+^ and HLA-A2^−^ AML patient samples. These data indicate the 8F4 bi-specific antibody robustly activates allogeneic T-cells following co-culture in a dose and PR1/HLA-A2-specific manner, and these activated T-cells exert their cytotoxicity toward local AML blasts.

Here, we determined the ability of the 8F4 bi-specific antibody to target and redirect T-cell cytotoxicity to lyse primary AML blasts. Leukapheresis samples from three HLA-A2 negative patients (#1–3) and four HLA-A2+ positive patients (#4–7) were co-cultured with healthy resting lymphocytes at a ratio of 2:1 (E:T) in the presence or absence of 8F4 bi-specific antibody for 18 h. Both CD4^+^ and CD8^+^ T-cells upregulated surface expression of CD69 in the presence of 8F4 bi-specific antibody when co-cultured with HLA-A2^+^ AML blasts, compared to HLA-A2^−^ AML blasts (Figure 6A). When patient samples were treated with 8F4 bi-specific antibody in the absence of allo-lymphocytes, there was a low level of T-cell activation detected on the autologous T-cells present in the sample (Supplementary Figure 1). There was no (or very low levels of) detectable CD69 on T-cells when co-cultured with primary AML blasts in the absence of 8F4 bi-specific antibody. The activated T-cells subsequently mediated lysis of CD33/34^+^ AML blasts (Figure 6B). Results show the 8F4 bi-specific antibody directs cytotoxicity of T-cells toward HLA-A2^+^ primary AML blasts at 200 pM, but shows minimal cytotoxicity of HLA-A2^−^ primary leukemia blasts at the same concentration.

## Discussion

This report presents the development and *in vitro* characterization of a therapeutic bi-specific antibody for the treatment of PR1/HLA-A2^+^ AML. This bispecific antibody simultaneously engages CD3 on resident T-cells and the PR1/HLA-A2 complex on the target cells and bring T cells in close proximity of target cells. Once engaged, CD3 cross-linking occurs, activating the T-cells to lyse nearby target cells via well-established cell-mediated cytotoxicity mechanisms. First, we showed successful eukaryotic expression and purification of the 8F4 bi-specific antibody. Specific target antigen recognition was demonstrated toward both CD3 and PR1/HLA-A2 with biochemical and cell-based methods. Following incubation of 8F4 bi-specific antibodies with T cells and PR2/HLA_A2^+^ target cells, we found that T cells were quickly activated, evidenced by upregulation of activation marker, CD69, and released various inflammatory cytokines such as Il-6, IFN-gamma, and TNF-alpha that can potentially augment anti-tumor immunity through apoptosis of tumor cells (23) Next, the bi-specific antibody showed robust redirected T-cell engagement and cytotoxicity toward PR1/HLA-A2^+^ AML cell lines and primary AML blasts was achieved at picomolar concentrations of bi-specific antibody.

Our *in vitro* results for the 8F4 bi-specific antibody are similar to those presented by others in this molecular class (10, 11). The 8F4 bi-specific antibody joins this class of bi-specific CD3-engaging antibodies to broadly activate T-cells in an antigen-specific manner, resulting in a safe cancer- targeting therapeutic. The potency of the 8F4 bi-specific antibody is high, highlighted by the low E:T ratios and picomolar antibody concentrations that resulted in detectable AML cytotoxicity. The potency of the 8F4 bi-specific antibody is comparable to others in this class of therapeutics (10, 12), and has proven to be adequate for successful treatment of patients (9). Of note, T-cells were activated in the absence of target antigen at high concentrations of bi-specific antibody, but this phenomenon can most likely be attributed to the high concentration of antibody leading to CD3 surface saturation and resultant cross-linking of surface CD3 and activation. This conclusion is supported by data which show that the reduction in 8F4 bi-specific antibody concentration is associated with a concomitant reduction in non-specific T-cell activation, while antigen-specific T-cell activation is maintained.

Pancytopenia, including a low lymphocyte count, is commonly seen in patients with AML, possibly limiting the therapeutic potential of immune approaches that rely on the patient's immune system. However, the ability of the 8F4 bi-specific antibody to mediate cytotoxicity at low E:T ratios, as low as 2:1 when using primary AML blasts as target cells, indicates that this therapeutic may be effective even in patients with a low lymphocyte count and a high leukemia burden. Recent studies examining the bi-specific engager AMG 330, which targets the leukemia antigen CD33, concluded that the *in vitro* activity of this agent correlated with the number of autologous T-cells present in patient leukemia samples, and that effective autologous T-cell redirected lysis could be detected at E:T ratios as low as 1 T-cell to more than 2,700 AML blasts. Interestingly, patients with newly-diagnosed AML were more responsive to AMG330 than those with relapsed/refractory disease for reasons yet to be identified (24). Similar to this study, we also detected activation of autologous T-cells in our experiments with primary patient samples, although at very low levels (Supplementary Figure 1). However, we examined cytotoxicity after 18 h following the addition of the 8F4 bi-specific antibody (vs. 48 h in the aforementioned study), which may not have allowed for complete T-cell activation and proliferation. In addition, AML patients with a high absolute lymphocyte count at the time of diagnosis were shown to have a decreased remission period and overall survival possibly due to a high number of regulatory T-cells in these patients (25). Although not directly tested here, the 8F4 bi-specific antibody may have the ability to overcome this dysfunctional component of the patients' immune system by targeting regulatory T-cells and converting them to functional cancer-fighting immune cells, as was demonstrated by other bi-specific antibodies (26, 27).

8F4 bi-specific antibody shares many characteristics with the T-cell engaging bi-specific antibody class of therapeutics, making it a strong candidate for the future treatment of HLA-A2^+^ AML. Additionally, this agent has potential therapeutic value as a treatment for solid cancers as well. Previously, we have shown that PR1/HLA-A2 is present on certain solid tumors such as breast cancer, lung cancer, and melanoma (14, 28, 29) via the mechanism of cross-presentation. This further expands a spectrum of diseases that can be treated with the 8F4 bi-specific antibody.

Further, the 8F4 bi-specific antibody represents the second antibody in a unique class of bi-specific T-cell engagers, in which the cancer-targeting region functions as a TCR-mimic, simultaneously recognizing the HLA molecule and its presented peptide. Another bi-specific antibody, the ESK1-BiTE, targeting the RMF peptide RMFPNAPYL produced from the intracellular oncogenic Wilms Tumor protein WT1 (30), has shown great promise in the treatment of several different malignancies *in vivo* and demonstrated a novel mechanism in which the bi-specific antibody recruits and activates autologous pre-existing T-cells in patients and facilitates a yet-to-be-defined mechanism aiding direct TCR-antigen interaction (11). In their report, *in vitro* treatment of an ovarian cancer patient sample with ESK1-BiTE generated an *in vitro* HER2/Neu-directed T-cell response. This was an unexpected finding as the ESK1-BiTE targets the WT1 antigen. The authors hypothesize this cytotoxic response is a re-activation of pre-existing T-cells targeting the HER2/Neu antigen due to the close proximity facilitated by binding of ESK1-BiTE to WT1 on the tumor and CD3 on the T-cell. It would be key to investigate this phenomenon further with both traditional bi-specific T-cell engagers and this sub-class of TCR-like bi-specific antibodies that target HLA-A2/peptide antigens. TCR-like antibodies characteristically have a high binding affinity and unique binding characteristics compared to traditional antibodies (17), and this may translate into unique binding properties of TCR-like bi-specific antibodies.

Bio-layer interferometry characterization of the 8F4 bi-specific antibody demonstrated lower affinity of the antibody for CD3 compared to PR1/HLA-A2, although the results of the *in vitro* analysis of bi-specific antibody binding to CD3 on the cell surface did not corroborate this finding. This disparity may have been due to the CD3 fusion protein that was used for the bio-layer interferometry experiments. Specifically, the bio-layer interferometry experiments employed a recombinant human CD3 delta^+^ CD3 epsilon protein, which is not a functional CD3 unit; the binding properties to this fragment may not be representative of true 8F4 bi-specific antibody binding to cellular CD3.

The lower CD3 binding affinity of the 8F4 bi-specific antibody, compared to its affinity to PR1/HLA-A2, it may be a benefit to its safety profile in patients. Studies with bi-specific antibodies targeting the breast cancer antigen HER2 demonstrated that a high affinity binding to CD3 may have a negative impact on the distribution of CD3-targeting bi-specific antibodies to the site of the tumor by promoting bi-specific antibody distribution to regions of the body high in CD3, such as lymph nodes (31). Lower affinity CD3 binding could provide two benefits: (1) prevention of localization of bi-specific antibody-bound T-cells to secondary lymphoid regions instead of to the tumor site, and (2) prevention of auto-reactive T-cell activation at high concentrations of antibody.

In summary, our data demonstrate that 8F4 bi-specific antibody exhibits potent antigen-specific redirected T-cell cytotoxicity against HLA-A2^+^ AML. This agent could potentially offer a much-needed therapeutic option in a patient population that lacks effective therapies. The 8F4 bi-specific antibody provides a novel immunotherapy to target PR1-presenting myeloid and solid tumor malignancies. Further investigations of the effects of the 8F4 bi-specific antibody *in vivo* are ongoing to better define the anti-leukemia activity and toxicity of this novel and promising immunotherapeutic.

## Ethics Statement

This study was carried out in accordance with the recommendations of the MDACC Institutional Review Board with written informed consent from all subjects. All subjects gave written informed consent in accordance with the Declaration of Helsinki. The protocol was approved by MDACC Institutional Review Board.

## Author Contributions

AH, JI, AS, HH, LS, KC-D, and JJM designed the experiments. AH, JI, PS, JM, SP, WR-V, and DZ performed the experiments. AH, JI, DZ, SL, AS, HH, GA, KC-D, JM, and JJM interpreted the results. AH, JI, GA, AS, LS, and JJM wrote the manuscript.

### Conflict of Interest Statement

JM and AS are inventors on a related patent and receive royalty payments. The remaining authors declare that the research was conducted in the absence of any commercial or financial relationships that could be construed as a potential conflict of interest.
