# Supplementary figures and images for: A Novel T-Cell Engaging Bi-specific Antibody Targeting the Leukemia Antigen PR1/HLA-A2
Source: Front Immunol. 2019 Jan 18;9:3153. doi: 10.3389/fimmu.2018.03153 (PMC6345694; doi:10.3389/fimmu.2018.03153)

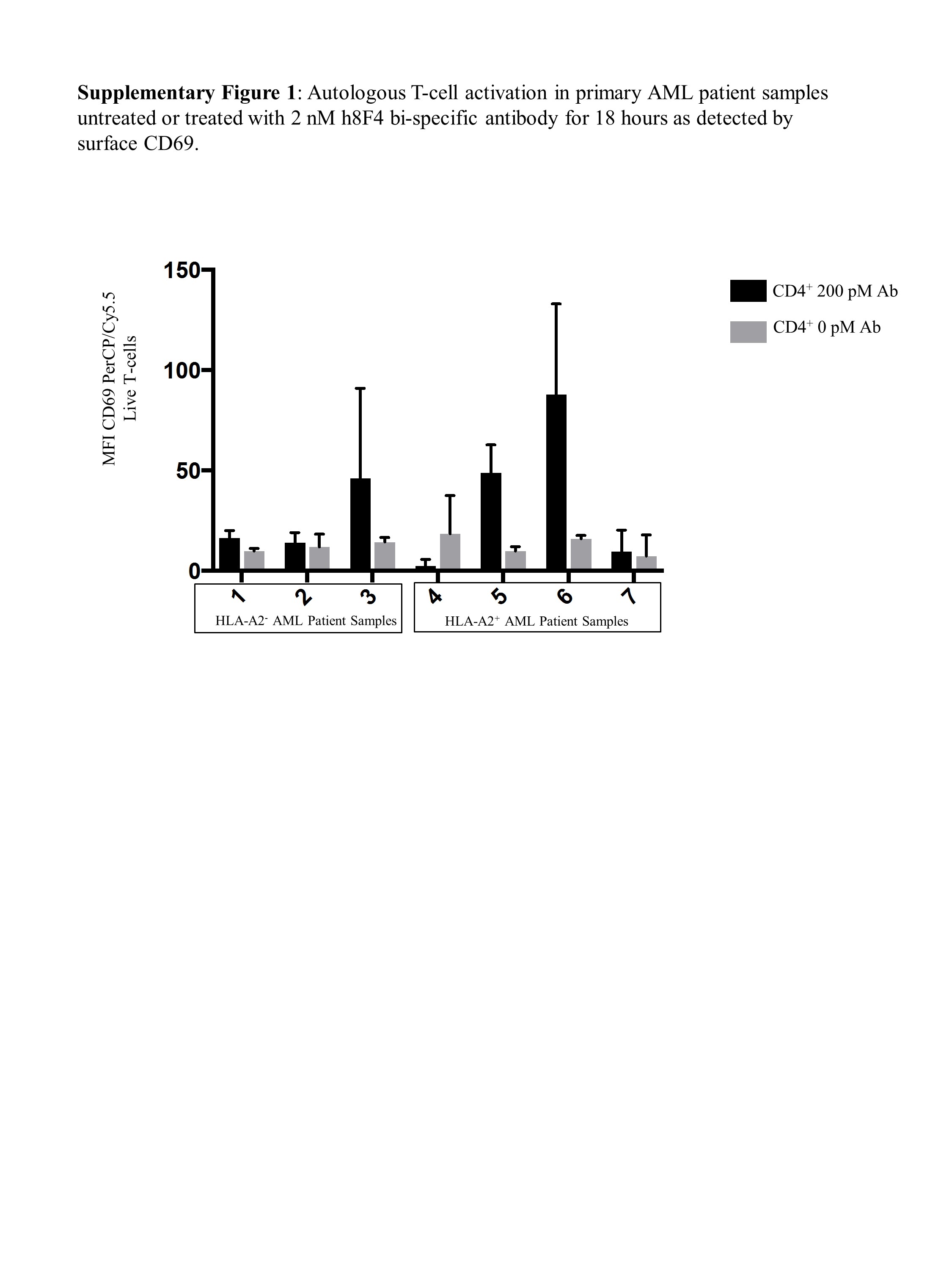

Supplement: Supplementary file 1 [file Image_1.JPEG]

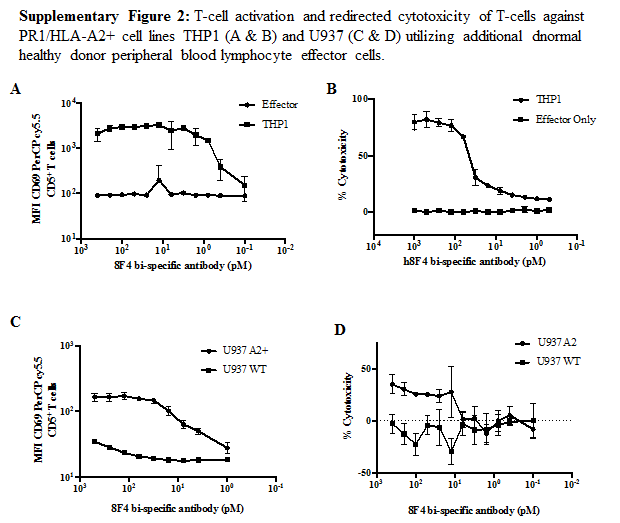

Supplement: Supplementary file 2 [file Image_2.TIF]
